# Supplementary material for: Are carbon emissions trading and green financial instruments synergistic? -Comprehensive quantitative research based on content analysis
Source: PLoS One. 2024 Mar 7;19(3):e0298601. doi: 10.1371/journal.pone.0298601 (PMC10919601; doi:10.1371/journal.pone.0298601)
Supplement: S1 Data — (ZIP) [file pone.0298601.s001.zip › Data/地级市Z_F工作报告.zip!/║■─╧/│╗╓▌╩╨/└┤╫╘╣1⁄2╓┌║┼í╛┬φ┐╦╩2╛▌═°í┐_2018.docx]

各位代表：

现在，我代表市人民政府向大会报告工作，请予审议，并请市政协委员及其他列席人员提出意见。

一、2017年工作回顾

2017年，在省委、省政府和市委的坚强领导下，在市人大、市政协的监督支持下，市政府紧密团结和依靠全市人民，坚持稳中求进工作总基调，大力实施“创新引领、开放崛起”“产业主导、全面发展”战略，以“三重”工作和四个“四年行动计划”为抓手，统筹稳增长、促改革、调结构、惠民生、防风险各项工作，取得了积极成效。预计（下同），全年实现地区生产总值2445亿元，同比增长7.8%；固定资产投资2618亿元，增长12.5%；社会消费品零售总额1004亿元，增长11%；全体居民人均可支配收入21534元，增长9%；8件具有长远影响的大事有6件基本完成，5个具有长远影响的重大基础设施项目前期工作扎实推进，“十大惠民工程”基本完成；人民群众安居乐业，社会大局和谐稳定。

——质量效益逐步提升。一年来，我们坚持以供给侧结构性改革为主线，以提高质量和效益为中心，全力以赴兴产业、强实体、提品质、增实效。有效供给不断增加。抓实“三去一降一补”重点任务，坚守安全、环保底线，“地条钢”等非法产能全面取缔，煤矿、非煤矿山、烟花爆竹等行业落后产能有序关闭退出。全市煤矿减少到70家，“十小”企业关停120余家，单位GDP能耗下降4%。房地产去库存任务提前一年完成。依法打击和处置非法集资，金融秩序稳定向好。实体经济加快振兴。出台并落实支持工业实体经济发展“22条”政策，强化金融对实体经济发展的支持，全市规模工业增加值增长7%、利润总额增长9%。电子信息产业加快壮大，高斯贝尔成功上市，东谷电子商务产业园投入运营。有色金属产业加快转型升级，26家企业进入全省有色金属行业50强，金贵银业成为全市首家百亿级企业。石墨新材料产业蓬勃兴起，南方石墨高碳石墨提纯、明大碳素等项目加快建设。出台并落实矿物宝石产业发展“10条”政策，宝玉石产业园加快建设，临武通天玉列入国家珠宝玉石名录。沃特玛新能源电池、东江湖大数据中心、万华生态板业等一批重点产业项目竣工投产。出台并落实园区发展“20条”政策，园区规模工业增加值增长8%，占全市规模工业增加值的78%。经济结构持续优化。农村一二三产业加速融合。粮食总产量192万吨。收购烟叶110.9万担。农产品加工销售收入、休闲农业营业收入分别增长15%、20%。农村电商蓬勃兴起，桂东县被评为全国电子商务进农村综合示范县。“郴州高山禾花鱼”获国家农产品地理标志登记保护，临武舜华鸭业荣获第五届“省长质量奖”。我市被列为全省食用农产品合格证管理和质量安全追溯体系建设试点市。国家服务业综合改革试点稳步推进。全域旅游示范区创建进展顺利。成功举办2017中国（湖南）红色旅游文化节和春、夏、秋季美丽乡村旅游文化节。接待游客人数、旅游收入分别增长20%、39%。我市被列为全国自驾旅游目的地试点城市。文化创意产业加快兴起，本土动漫《小邑时空传》在央视播出。会展经济加速发展，成功举办第五届矿博会、第三届农博会、2017国际篮联亚洲冠军杯等一系列重大展会和体育赛事。湘南国际物流园成功创建国家级示范物流园区。第三产业增加值增长10.7%。财政收入质量提升。出台并落实加强财源建设“12条”政策，财政增收后劲不断增强。我市被评为全国百佳国税地税合作市级示范区。全市完成一般公共预算收入203.3亿元、地方财政收入130.5亿元。税收占一般公共预算收入的74.7%，比上年提高18.9个百分点；非税收入占地方财政收入的39.5%，比上年降低19.9个百分点。发展动能在产业主导中加速蓄积，实力郴州建设迈出了新的步伐。

——改革创新全面深化。一年来，我们坚持向改革要活力、向创新要动力，推动经济发展质量变革、效率变革、动力变革。全面深化改革落地见效。“放管服”改革深入推进，清理规范市本级164项行政审批及其他行政职权涉及中介服务事项，第二批行业协会商会脱钩试点基本完成。政务服务“一次办结”改革强力推进，减证便民专项行动稳步开展。公共资源交易改革成效初显。不动产交易登记一体化改革、城市执法体制改革稳步推进。土地登记确权颁证工作基本完成。土地流转、林地“三权分置”等农业农村改革成效明显。供销社综合改革有序推进。财税体制改革纵深推进，财政预决算公开、国库集中支付等改革走在全省前列。探索建立“三定三明三到位”政府债务监管机制，政府性债务风险得到有效防控。投融资体制改革不断深化，民间投资占全市固定资产投资的65%。市城投集团“转型、化债、求效”步伐加快。医药卫生体制改革、文化市场改革、事业单位分类改革等稳步推进。出租汽车行业改革成功实施。军民融合迈出新步伐，郴州战备钢桥保障基地成立。创新驱动发展成效初显。创建国家可持续发展议程创新示范区工作积极推进。石墨精深加工、纳米高纯铋粉、银系催化剂等领域技术攻关取得突破。南方新型碳材料研究院、国家超级计算长沙中心（郴州）分中心、湖南大学和中南大学技术转移中心（郴州）分中心等新型研发平台启动建设。获批建设有色金属固体废弃物回收利用、微晶石墨两个全国专业标准化技术委员会机构。出台并落实引进企业高层次人才“22条”政策，郴籍科技专家库加快完善。国家知识产权示范城市创建工作扎实有效。专利授权量增长15%，发明专利增长50%。格兰博智能家居、星源智能装备制造等一批创新型企业加快成长。新增高新技术企业50家。市场主体不断发展壮大。实施“多证合一、一照一码”登记制度改革，允许“一址多照”“一照多址”和省级以上园区企业集群注册，市场主体服务便利化系统性改革获省深改组好评。培育各类新型农业经营主体近4万家。全市实有市场主体总数、注册资本（金）分别增长13.9%、22%。发展活力在创新引领中加快迸发，创新郴州建设取得了新的突破。

——开放崛起势头强劲。一年来，我们认真实施“五大对接行动”，全省开放崛起“排头兵”地位加快形成。精准招商有力有效。成功承办第八届湘商大会和第五届湘南投洽会等系列重大招商经贸活动。坚持以产业链为主导，精准对接500强、对接湘商会，积极“引老乡回故乡建家乡”，签约市级以上重大产业项目133个，计划总投资1452.15亿元。全市实际利用外资16.8亿美元、到位内资526亿元，分别增长10.5%、12.3%。成功引进香港清水湾机器人投资公司、德国科德宝集团来郴建设生产（示范）基地，引进中国物流、中国农批、中国正信集团等一批央企战略投资，白银精深加工和供应链、3D电子玻璃生产、莽山文化旅游产业开发、碧桂园龙女康养旅游特色小镇、华谊兄弟电影小镇等一批优质产业项目成功落户。构建产业联盟合作机制，沃特玛新能源汽车创新联盟苏仙产业园、湖南广东家居智造产业园、东江湖大数据产业园等特色园区初步形成产业集群。开放平台加快完善。郴州综合保税区通过验收。“跨境一锁”快速通关系统正式运行。中国（湖南）自贸区郴州片区申报工作有序推进，自贸试验区经验复制推广任务基本完成。郴州进口肉类指定查验场加快建设。出口食品农产品质量安全示范区加快创建。外经外贸增长迅速。外贸“破零倍增”计划成效明显，完成外贸进出口总额35.3亿美元，增长30.2%。积极对接“一带一路”，7家本土企业获境外投资许可。对外承包工程和劳务合作营业额增长33.9%。坚持用最优的生态环境拥抱粤港澳大湾区、用最优的营商环境拥抱海内外客商，开放郴州建设呈现出新的活力。

——城乡环境品质提升。一年来，我们坚持绿色发展、协调发展，尊重自然、顺应自然、保护自然，促进人与自然和谐共生，城乡环境更加美丽宜居。基础设施更加完善。市中心城区实施基础设施项目198个，完成投资120亿元。高铁西站扩容提质、京港澳高速郴州北互通等项目竣工，东江引水工程（一期）可在春节前具备试通水条件，北湖机场、东江引水工程（二期）等重大项目加快推进。岳汝高速公路与广东段对接通车，建设干线公路160公里，改造农村公路1741公里、危桥46座，完成农村公路安保工程1193公里。“治超”和城乡客运公交化改造持续推进。嘉禾县入选全国城乡交通运输一体化示范县首批创建县。农田水利设施加快完善，桃源水库竣工蓄水，茶安水库提质扩容项目加快推进。嘉禾县城乡供水一体化智慧管理改革模式全国推介。城镇品质持续提升。国家新型城镇化综合试点加快实施。“两供两治”“两房两棚”工作扎实推进。城市综合管廊、“海绵城市”建设稳步开展。完成垃圾收集处理设施建设投资7.9亿元，整治黑臭水体62个。加快建设“公交都市”，绿色公交比率达78%。“气化郴州”步伐加快，新增管输天然气用户3.6万户。扎实开展创建全国文明城市工作，顺利通过国家卫生城市复审和国家园林城市复查，成功创建国家节水型城市，全市涌现出全国文明单位4个、全国文明村镇6个、全国文明校园2所。县城和小城镇建设加快提质。资兴市、桂阳县成功创建国家园林城市（县城）。热水镇获评全国文化特色小镇“十佳样本”。美丽乡村加快建设。出台并落实鼓励市民下乡建设美丽乡村“22条”措施，启动实施美丽乡村四年行动计划，“十城百镇千村”新农村建设工程深入实施，农村村民建房更加规范。新增省级美丽乡村示范村6个、市级美丽乡村示范村30个、小康新村150个。永兴县被列为全国第一批农村生活垃圾分类和资源化利用示范县，板梁村被评为全国生态文化村。资兴市获评中国美丽乡村建设示范市。马田镇、行廊镇成为第三批湖南省美丽乡镇示范镇。北湖区三合村被评为“中国美丽乡村百佳范例”。落实最严格耕地保护制度，连续18年实现耕地占补平衡。环境治理成效明显。以中央环保综合督察为契机，切实解决了一批突出环境问题。积极创建空气质量达标示范城市，空气质量优良率91.9%。认真落实湘江保护和治理第二个“三年行动计划”，“一湖两河三江”、三十六湾等重点区域综合治理加快推进。12个饮用水源地水质100%达标，6个国家考核断面、38个地表水考核断面水质达标率分别为100%、97.4%。“河长制”全面推行，全国水生态文明城市建设试点通过验收。完成造林27.5万亩，森林覆盖率67.73%。永兴县列入国家首批采煤沉陷区综合治理试点县。宜章赤石国家石漠公园通过评审。美丽家园在绿色发展、协调发展中焕发新姿，生态郴州建设探索出新的路径。

——发展成果更加惠民。一年来，我们认真践行以人民为中心的发展思想，一大批惠民政策和举措落地见效。精准脱贫扎实推进。“四跟四走”产业扶贫成效明显，小额扶贫信贷余额突破19亿元，创建全国“就业扶贫基地”4家。82个易地扶贫搬迁安置项目进展顺利。农村低保清理整顿取得阶段性成效，“两线合一”全面落实。245个贫困村预脱贫退出，9.1万贫困人口预脱贫销号，桂东县脱贫“摘帽”取得阶段性成果。民生短板加快补齐。财政民生支出298.9亿元，占一般公共预算支出的77.6%。省定民生实事项目全面完成。新增城镇就业7.5万人、农村劳动力转移就业4万人，城镇登记失业率3.48%。完成棚户区改造3.4万户、农村危房改造2.1万户。建设（改造）农村综合服务平台249个、贫困地区文化综合服务中心示范点18个。各项事业协调发展。全民参保登记计划任务全面完成，城镇居民医保和新农合整合到位，机关养老保险改革跨省异地结算试点有序推进。城乡义务教育一体化发展统筹推进，乡村教师支持计划有效落实。全市新增教师编制2954个，县城以上城区新增义务教育学位4万个，市城区化解“大班额”三年行动计划成效明显。在全省率先推行普通高中免学杂费政策。职业教育内涵发展进程加快。湘南学院、郴州职业技术学院、郴州技师学院、湘南幼专加快发展。医药卫生体制改革深入实施，公立医院综合改革实现全覆盖。市城区医疗卫生服务发展三年行动计划有序推进。爱国卫生运动成果有效巩固。疾病防控和母婴安全保障有力有效，新生儿出生缺陷发生率明显下降。中医药事业稳步发展。依法稳妥实施“全面两孩”政策，计生工作保障机制更加完善，基层基础更加牢固，服务关怀更贴近民生。公共文化服务体系更加健全。文化遗产和传统村落保护成效明显。国防动员、人民防空、民兵预备役、双拥共建工作取得新成绩。对台、外事、侨务、老年人、气象、史志、档案、保密、统计、防震减灾、妇女儿童、民族宗教、慈善、残疾人、红十字会等工作取得新进展。社会大局和谐稳定。“法治郴州”“平安郴州”建设不断加强。深入开展“三打两治一清”行动，刑事案件案发率下降27.6%，破案率上升5.7%。资兴市再获全国社会综合治理“长安杯”。桂阳县人民调解“百米服务圈”经验全国推介。村（居）委换届圆满完成。退役军人服务保障进一步完善。严格落实安全生产“三个责任清单”和重大事故隐患治理“一单四制”，安全生产形势总体平稳。应急管理、信访维稳、防汛抗旱、森林防火、消防救援、防灾减灾、食品药品监管等工作成效显著。人民生活在共享发展中越来越好，人本郴州建设取得了新的进展。

——自身建设不断加强。一年来，我们树牢“四个意识”，突出转政风、抓执行、强落实，着力建设忠诚、法治、办事、高效、廉洁政府。推进“两学一做”学习教育常态化制度化，严肃各级政府及其部门党内政治生活，市政府班子凝聚力不断增强。认真执行市人大及其常委会的决议决定，自觉接受人大和政协监督。办理人大代表建议323件、政协提案276件，满意和基本满意率100%。贯彻《法治政府建设实施纲要（2015-2020年）》，健全完善市政府工作规则和重大行政决策程序规定，落实政府法律顾问和市政府常务会议学法制度，政府科学决策机制不断完善，行政权力运行进一步规范。强化市长公开电话投诉案件跟踪办理，开展“下基层、找问题、解难题、增效益”“抓项目、促增长、提质效、保稳定”等系列活动，依法有效化解社会矛盾纠纷，着力解决了一批群众最关心最直接最现实的利益问题。发挥各民主党派、工商联、无党派人士的参政议政作用，支持工会、共青团、妇联等群团组织充分发挥桥梁纽带作用，引导志愿服务组织发挥公益作用，最广泛地凝聚起共推发展的强大力量。不断拓展和强化审计监督、财政评审，财政资金使用和政府性投资效益持续提升。深入开展“治陋习、树新风”深化作风建设年活动，着力构建“亲”“清”新型政商关系，扎实推进“雁过拔毛”式腐败问题专项整治，以“零容忍”态度惩治腐败，风清气正的政治生态进一步巩固提升。政府执行力、公信力在“五个郴州”建设中不断强化，展示了服务型政府新的形象。

各位代表！发展道路不会一帆风顺，宏伟目标不会一蹴而就，必须要有“功成不必在我，建功必须有我”的胸襟和担当，努力把基础打得更牢，把步伐迈得更稳，把质量提得更优，把成效做得更实。一年来，我们立足长远兴产业、强实体，将重心放在补链建链强链上，一批具有长远影响的产业项目落地实施；我们顶住压力调结构、提质量，舍弃短期低质、低效的快速度，换取更高质量、更有效率、更加公平、更可持续的发展；我们突出重点补短板、强弱项，更加注重“微建设”“微服务”，努力提升人民群众的获得感幸福感安全感。这些思路和举措，顺应了新时代新变化，践行了新理念新方略，既为郴州长远发展奠定了坚实基础，也为郴州当前变化赢得了广泛认可：水生态文明建设、县域内城乡义务教育一体化发展、家庭医生签约服务、公立医院薪酬制度改革、美丽乡村建设、高标准农田建设、供销社综合改革、光伏扶贫、畜禽水产品质量安全追溯、农村集体资产股份权能改革、水利建设管理、公安“三最”便民利民服务等“郴州经验”“郴州样本”在全省乃至全国推介。这些成绩的取得，是市委坚强领导、科学决策的结果，是市人大、市政协监督支持、积极参与的结果，是全市人民奋力拼搏、尽责担当的结果。在此，我代表市人民政府，向全市各族人民，向人大代表、政协委员，向各民主党派、工商联、无党派人士和各人民团体，向离退休老同志，向驻郴部队官兵和中省驻郴单位，向所有关心支持郴州改革发展的各级领导、各界朋友，表示衷心的感谢并致以崇高的敬意！

同时，我们也清醒看到政府工作存在的困难和不足，主要是：科技创新能力不强，发展动能转换不快，经济可持续发展和财政可持续增收基础不牢；环境污染历史存量削减难度增大，突出环境问题仍然较多；城乡、区域发展不平衡不充分，农民持续增收难度较大，脱贫攻坚任务艰巨；重点领域、重点行业安全隐患和风险突出，安全生产形势仍然严峻；部分干部的能力素质、狠抓落实的本领、经济工作的精准化精细化水平有待提升；一些地方和部门不正之风和腐败现象仍有发生，“四风”问题反弹回潮仍然存在，反腐败斗争形势依然严峻复杂。对此，我们将以高度负责的态度，采取更加积极有效的措施，切实加以解决。

二、2018年工作安排

2018年是贯彻党的十九大精神的开局之年，是我国改革开放40周年，是决胜全面建成小康社会、实施“十三五”规划承上启下的关键一年。2018年政府工作的总体要求是：以习近平新时代中国特色社会主义思想为指导，全面贯彻落实党的十九大精神和中央、省委、市委经济工作会议精神，加强党对政府工作的领导，坚持依法行政，继续坚持稳中求进工作总基调，坚定不移贯彻新发展理念，紧扣社会主要矛盾变化，按照高质量发展的要求，统筹推进“五位一体”总体布局，协调推进“四个全面”战略布局，着力推进供给侧结构性改革，突出抓重点、补短板、强弱项，强力实施“创新引领、开放崛起”“产业主导、全面发展”战略，持之以恒“兴产业、强实体、提品质、增实效”，统筹推进稳增长、促改革、调结构、惠民生、防风险各项工作，在打好防范化解重大风险、精准脱贫、污染防治的攻坚战方面取得扎实进展，确保郴州经济更好、更实、更协调、更可持续发展。

主要预期目标是：地区生产总值增长8%左右；规模工业增加值增长7.5%左右；固定资产投资增长11.5%左右；社会消费品零售总额增长11%左右；一般公共预算收入增长7%以上；外贸进出口总额增长18%左右；实际利用外资增长10%左右；全体居民人均可支配收入增长9%左右；城镇登记失业率控制在4.5%以内。

围绕实现上述目标，要突出抓好九个方面的工作：

（一）始终保持战略定力，着力推进经济可持续发展。坚持以供给侧结构性改革为主线，加快推动经济发展质量变革、动力变革、效率变革。扎实抓好“三去一降一补”。不折不扣完成煤炭、造纸、采选冶炼等重点领域落后、过剩及不安全产能退出任务，着力优化存量资源配置，扩大优质增量供给。全面落实各项企业减负政策，促进企业降本增效。完善促进房地产市场平稳健康发展的长效机制。服务于供给侧结构性改革这条主线，促进形成金融和实体经济、金融和房地产、金融体系内部的良性循环，加快创建金融生态良好城市。做好重点领域风险防范和处置，坚决打击违法违规金融活动，严控政府性债务规模，优化政府性债务结构，做好财政金融风险监测评估，打好防范化解重大风险攻坚战。集中全力发展实体经济。把发展经济的着力点放在实体经济上，实施“小微企业成长计划”“领军工业企业培育计划”，“一企一策”开展企业帮扶。加强企业上市工作，支持上市公司实施产业整合，努力培育一批竞争力、带动力较强的大企业大集团。充分发挥企业市场主体作用，促进资源要素高效配置，增强市场机制内生动力。弘扬优秀企业家精神，大力发扬工匠精神。切实做大做强产业园区。深入落实产业园区发展“20条”政策，突出集约节约、质量效益，进一步完善园区发展考核指标体系。加快推进特色园区发展，重点推动郴州高新区新能源汽车产业园、永兴循环经济园、湖南广东家居智造产业园、宜章氟化学工业集中区、宝玉石产业园等做大做强。坚持产城人融合，加快完善园区生活配套设施和服务。力争园区规模工业增加值增长7.5%，占全市规模工业增加值的比重提高到80%。积极推动资兴经开区、郴州经开区创建国家级经开区。推进产业融合协调发展。大力实施“互联网+”计划，加快新一代信息技术与各行业深度融合。大力加强物流园区建设，着力引进和培育现代物流龙头企业，积极推进供应链创新应用，加快发展第三方物流，促进生产性服务业与制造业融合发展。引导和扶持农产品精深加工，创建一批休闲农业示范园和田园综合体。促进“五大融合”，加快发展健康产业。实施“一村一网店”工程，大力发展涉农电商。抓实文化旅游体育千亿产业四年行动计划，全面实施旅游“三大工程”，开展第四批全国旅游标准化试点示范工作，引导扶持精品民宿发展，办好郴州国际生态休闲旅游文化节，积极拓展海内外旅游市场，加快全域旅游发展。力争接待游客人次、旅游总收入分别增长15%、25%。充分发挥矿博会的平台和品牌作用，坚持“六化”方向，办好第六届矿博会等一批特色会展和重大体育赛事。力争服务业增加值增长11.1%。

（二）推动产业转型升级，着力提升发展质效。围绕构建富有郴州特色的现代化经济体系，扎实推进产业转型升级。全力抓好产业建设“六个一”工程。对接落实全省“产业项目建设年”活动和“五个100”，深入实施四个“四年行动计划”，努力实现新增1个千亿园区（资兴经开区）、新增1个500亿产业（文化旅游产业）、新开工10个投资50亿元以上的重大产业项目、新引进10个500强企业、新实施100个科技创新项目、新引进100名高层次人才的目标。着力推进传统产业转型发展。实施优势产业链培育计划，完善有色金属“五个一战略体系”，着力建设好有色金属固体废弃物回收利用、微晶石墨等全国专业标准化技术委员会机构。充分发挥好钨、铋、石墨、萤石等资源储量在全球的绝对优势，重点培育有色金属、石墨新材料、矿物宝石、氟化工、碳酸钙等产业链，促进优势产业迈向全球价值链中高端，加快把郴州打造成世界铋产业基地、钨产业基地、石墨产业基地、氟化工产业基地。支持永兴县金银及稀贵金属产业、嘉禾县锻铸造产业整合升级。加快建材、食品、医药等传统产业提质。加快发展现代特色农业，抓好绿色农产品标准化生产基地、外向型农产品生产基地建设，全力打造粤港澳大湾区优质食材基地。确保粮食总量稳定和粮食安全。严格按计划、按合同组织烟叶生产，着力提高烟叶质量，促进烟农稳定增收。着力发展新型特色产业。积极对接“中国制造2025”，加快培育人工智能、新材料、新能源、电子信息、先进装备制造等战略性新兴产业。依托东江湖大数据产业园，积极发展大数据产业，加快把郴州打造成华南地区“绿色数据谷”。落实支持通航产业发展“8条”政策，推进通航产业发展和临空经济区规划建设，加快建设通用航空试点城市。力争战略性新兴产业增加值占GDP比重达20%以上。鼓励发展高科技农业及会展农业、农业众筹等农业新业态。推动产业绿色低碳循环发展。加快发展绿色生态产业，持续推进循环经济示范建设，努力打造全国工业资源综合利用示范基地。推进绿色矿业发展示范区建设，促进矿产资源高效利用，做强做优矿业经济。继续推进国家低碳城市试点，壮大节能环保、清洁生产、清洁能源、装配式建筑等产业，抓好风电、光伏发电等项目建设，推进资源全面节约和循环利用。推进节能减排，单位GDP能耗下降3.4%，完成省下达减排目标。增强投资对产业发展的保障。主动引导资金投向，着力优化投资结构，把更多的资金投入到产业发展上来，确保产业投资在全市固定资产投资中占比达到60%以上。改革政府投资方式，加快组建产业引导股权投资基金，有效支持中小企业和优势产业、新兴产业发展。鼓励金融创新，强化政银企对接合作，做强担保公司，着力缓解融资难、融资贵、融资慢等问题。支持有实力的企业设立产业发展基金，吸引社会资金投入政府重点支持的产业，确保民间投资在全市固定资产投资中占比提高到80%以上。支持企业提升核心竞争力。鼓励企业加大技术改造力度，引导支持企业采用先进适用技术，提升企业设计、制造、工艺、管理水平。大力支持企业质量品牌建设，促进企业提升知识产权管理水平，引导和鼓励企业在基础性、前瞻性、战略性产业领域建设具备先进水平、服务产业发展的创新基础设施，促进企业占领行业技术高地。

（三）扎实抓好项目建设，着力扩大有效需求。充分发挥内需对经济增长的支撑作用，着力抓实项目投资特别是产业投资、工业投资，着力培育消费热点，促进投资、消费良性互动。推进重大项目建设攻坚。全市初步安排总投资1亿元以上重大建设项目615个，年度计划投资1157亿元。重点抓好沃特玛新能源汽车产业园、郴州卷烟厂易地技改、中国供销郴州农副产品物流园、桂东县全域旅游PPP等352个重大产业项目；北湖机场、人民东路延伸段、莽山水库、中石化新疆煤制气外输管道（郴州段）、衡武高速桂阳连接线、G107良田绕镇线等135个重大基础设施项目；东江湖生态保护、三十六湾重金属污染治理、嘉禾县黑臭水体治理等25个生态环保项目；东江引水工程（二期）、市一中新校区等103个重大民生和社会事业项目。落实重大项目“五个一”“一单五制”工作机制，完善项目建设责任体系，健全重点项目联动协调、快速反应机制，着力优化项目施工环境。依法推进征地拆迁、杆（管）线搬迁等工作，强化项目建设用地、资金等要素保障，破解制约项目建设的瓶颈问题。继续抓好赣郴永兴铁路、郴资永轨道交通等重大项目前期工作，谋划储备一批重大项目。强化重大项目稽查和政府投资项目审计，做到投资专项监管全覆盖。努力扩大消费需求。深入推进“互联网+商贸流通”行动，促进线上线下融合发展，推进商贸流通转型升级。实施“边贸市场促进工程”“县乡流通再造工程”，完善流通基础设施。发展健康养老、家政服务等新兴消费。进一步扩大和升级信息消费。加快解决房地产领域历史遗留问题，稳定住房消费。巩固汽车等大宗消费。强化消费市场监管，营造放心消费环境。

（四）全面深化改革创新，着力增强发展动能。坚持以改革创新推动发展方式转变、新旧动能转换，把各领域改革推向纵深。抓实改革措施落地。继续深化“放管服”改革，推进“互联网+政务服务”，构建政务服务新模式。深化商事登记制度改革，全面实施“多证合一”，在郴州高新区试点推行“证照分离”。深化投融资体制改革，进一步激发社会投资活力。分类创新政府资源配置方式，建立公共资源目录清单，加强公共资源交易监管，推动资产配置实现效益最大化和效率最优化。稳步推进市城市规划区地下停车位产权办理工作。深化户籍制度改革，推动农业转移人口和其他常住人口在城镇落户。巩固和完善农村基本经营制度，深化农村土地制度改革，完善承包地“三权”分置制度，稳妥推进集体产权制度改革、国有农场改革。坚持科技创新驱动。深化科技体制改革，加快建立以企业为主体、市场为导向、产学研深度融合的技术创新体系。深化与大企业大集团、高校、科研院所的合作，推动产业技术研究项目建设，重点推进与湖南大学、中南大学产学研合作项目的落地。落实鼓励和推进科研成果转化“16条”政策，加快推进企业、政府、高校联合的成果中试转化体系建设，加强对中小企业创新的引导、鼓励和支持。加快南方新型碳材料研究院建设。跟踪中国五矿在郴建设世界一流的钨研发中心。支持依托市内骨干企业组建世界铋业协会、铋产业研究院。全面推进创新创业园区发展，加快创建国家可持续发展议程创新示范区、国家知识产权示范城市。大力培育高新技术企业，力争高新技术产业产值增长15%。推进人才强市战略。落实引进企业高层次人才“22条”政策，建立健全人才资源库，确保人才引得进、用得好、留得住。完善校企合作机制，支持在郴高校、职（技）校应用学科和特色专业建设，促进教育链、人才链和产业链、创新链有机衔接。推进质量强市建设。大力实施品牌战略，推进质量诚信体系建设，开展质量提升行动，实施增品种、提品质、创品牌工程，打造一批“郴州智造”“郴州创造”品牌。加快完善农产品质量安全监管追溯体系建设。加强农业品牌建设和资源整合，提升郴州农产品品牌知名度和市场占有率。

（五）突出招商引资引智，着力加快开放崛起。充分发挥湖南向南开放“桥头堡”的优势，着力打造“向南开放看郴州”的品牌。抓实抓好精准招商。大力实施“对接500强、提升产业链”“对接湘商会、建设新家乡”和“对接北上广、优化大环境”专项行动，更好地发挥产业招商小分队和驻外招商机构“精、专、准”优势，围绕“五大重点产业”，着力引进一批总部型、引领型、补链型、核心型的产业提升项目和高附加值的大项目、好项目，提升招商引资质效。主动对接“长三角”，积极组织参加2018湖南“沪洽周”等经贸活动。精心筹办好湘南投洽会和郴商大会等招商活动。推进开放平台升级。加快进口肉类指定查验场、京港澳高速公路宜章口岸作业区、湖南对接粤港澳仓储物流基地建设，推进郴州口岸与广东口岸、香港口岸无缝对接，积极申报郴州高铁客运口岸，促成广州港集团等国际物流龙头企业参与郴州内陆港（铁路口岸）投资运营。继续抓好中国（湖南）自贸区郴州片区申报工作。推进国际贸易“单一窗口”共享数据标准化。加强与沿海、沿边通关协作，积极推行“联合查验、一次放行”通关新模式，推动检验检疫一体化。深化拓展开放合作。积极探索开放合作新模式，主动加强与粤港澳大湾区城市对接合作，深化与“红三角”地区的经贸合作。实施“对接一带一路、推动走出去”专项行动，重点培育金旺铋业、高斯贝尔、金贵银业、杉杉科技、华磊光电等“走出去”骨干企业。鼓励企业抱团参与国家援外项目招投标，重点依托郴电国际大力发展对外工程承包，拓展国际工程承包市场。促进对外贸易转型。继续实施“破零倍增”计划，培育一批外贸综合服务企业、园区外贸综合服务中心，提升外贸综合服务能力。大力拓展外贸新业态、创新新模式，着力引进一批外向型实体龙头企业和加工贸易项目。加快提升郴州综合保税区功能，充分发挥其服务开放型经济发展的龙头作用。积极创建郴州出口食品农产品质量安全示范区，扩大食品农产品出口。切实优化发展环境。深化政务服务“一次办结”改革，加强集中审批、并联审批，优化事前服务，加强事中、事后监管，进一步提升行政效能和政务服务水平。树立法治环境是核心竞争力思想，深入推进“法治郴州”建设。推进创建国家社会信用体系建设示范城市工作，打造“诚信郴州”品牌。深化“亲”“清”新型政商关系，大力营造企业家健康成长环境，促进非公经济加快发展。建立优化大环境投诉举报和协调联动机制，严肃查处损害经济发展环境的行为。

（六）提升城乡生态品质，着力建设美丽家园。认真践行“绿水青山就是金山银山”理念，扎实推进生态郴州建设四年行动计划，推进实施“厕所革命”三年行动计划，切实打好环境治理攻坚战，使主要污染物排放总量大幅减少，生态环境质量总体改善。着力解决突出环境问题。抓实湘江保护和治理第二个“三年行动计划”年度任务。加强东江湖流域生态环境保护，助推建立东江湖生态补偿机制。全面落实“河长制”，强化源头防控和属地责任，扎实推进重点流域水污染治理，确保各监测断面和饮用水源地水质稳定达标。加强渔政执法，严厉打击电鱼、毒鱼等违法犯罪行为。强化工业集中区污染处理设施建设，确保全市所有省级及以上工业园区配套污水处理设施全部建成并联网运行。推进“排污费”改征“环保税”顺利实施。抓好污染源普查，继续推进工业污染源全面达标排放、饮用水源地环境保护等专项行动。切实抓好农业面源污染防治。落实最严格的水资源管理制度，确保实现“三条红线”管控目标。建立大气污染源排放清单，编制大气环境质量达标规划，持续推进空气质量达标示范城市创建，确保市城区空气优良率90%以上。加强自然生态保护工作。完善生态保护红线划定后续工作，抓好生态保护红线勘界定标，实行定期精准巡查，严格生态保护红线和自然保护区监管，确保全市整体生态环境安全。引导各方面资金投入，培育一批专门从事生态保护修复的专业化企业，实施好生态保护修复重大工程。全面推进森林质量精准提升，完成人工造林25万亩，封山育林30万亩。坚持土地数量、质量、生态“三位一体”保护。推广安仁县城乡建设用地增减挂钩经验。做好第三次全国土地调查工作。贯彻实施乡村振兴战略。坚持农业农村优先发展，完善农村基础设施网络，扎实抓好水利“两个建设、三项工程”，抓实洁净乡村四年行动和农村“双改”三年行动计划，继续抓好农村环境综合整治整县（市、区）推进工作，健全农村基本公共服务体系，加快推进美丽乡村建设，重点打造西河沿线城乡融合发展示范带。大力培育新型农业经营主体，引导土地有序流转，发展适度规模经营的特色产业，促进农民持续增收。坚持自治、德治、法治相结合，传承农村优秀传统文化，大力倡导文明乡风。切实提升城镇建管水平。强化规划引领和城市设计，加强专项规划、详细规划编制和重点区域的城市设计。加强新一代信息技术在城市建设管理中的应用，稳步推进“智慧城市”建设。突出以“双修”为重点的基础设施建设，推进地下综合管廊试点、“海绵城市”建设和黑臭水体整治。加快实施城市垃圾无害化处理和综合利用项目。加强城市提质改造，提升城市品质和文化内涵。着力打通一批“断头路”，完善城市道路网络，优化城市交通组织，加快实施智能停车系统和立体停车库建设，有效缓解城区“行车难”“停车难”问题。加快推进“公交都市”创建，争取绿色公交比率达85%。持续巩固 市城区“限摩规电”成果，以就近入学、就近诊疗、健身休闲、社区警务、便民服务为重点，着力打造“一公里公共服务圈”。坚持重心下移，按照责权利相统一原则，进一步理顺城管体制机制。坚持为人而创、为城而创、为郴州发展而创，做到班子不散、精力不散、队伍不散、人员不散，锲而不舍推进全国文明城市创建工作。切实抓好国家新型城镇化综合试点，全面加强县城和重点镇、特色镇等小城镇建设管理，扎实推进以人为核心的新型城镇化。支持桂阳撤县设市。

（七）更加注重精准施策，着力推进脱贫攻坚。坚持把脱贫攻坚作为第一民生工程来抓，按照“一超过、两不愁、三保障”标准，精准落实帮扶措施，保证现行标准下的脱贫质量，确保实现桂东县“摘帽”，汝城县、安仁县、宜章县实现整体脱贫，剩下129个贫困村全部退出，贫困发生率降至2%以内，坚决打赢脱贫攻坚战。发展特色产业。立足帮助贫困人口可持续增收，坚持“四跟四走”，因地制宜发展特色产业，加快培育农业龙头企业，解决好特色农产品市场和销路问题。大力发展乡村旅游，吸纳贫困农民就近就业。完善委托帮扶风险防范机制，促进扶贫小额信贷健康发展，帮助贫困户直接增收。抓好易地搬迁。坚持因地制宜、科学规划，充分尊重贫困户个人意愿，实行集中与分散相结合、以小规模集中安置为主的搬迁模式，做到易地搬迁与产业发展并举、安居与乐业并重、搬迁与脱贫同步，确保贫困人口搬得出、稳得住、能发展。全面完成第二批、第三批全国大中型水库移民避险解困试点工作任务。推进“四好农村路”建设。聚焦突出问题，完善政策机制，注重发挥基层和农民的积极性，有效提升农村公路建设、管理、维护、运营水平，助推脱贫攻坚和乡村振兴。落实精准帮扶。精准落实覆盖各级各类教育的学生资助体系，实现建档立卡贫困户子弟上学扶助全覆盖，确保不让一个学生因家庭经济困难而辍学。继续抓好精准扶贫“三包式”技能培训，推动农村劳动力转移就业向贫困地区、贫困人口倾斜。加大健康扶贫力度，实施“三个一批”行动计划，有效防止因病致贫、因病返贫。精准落实政策兜底，关爱丧失劳动能力的贫困户。加强贫困村驻村工作队选派管理和考核激励，确保帮扶精准、群众满意。激发内生动力。坚持扶贫先扶志，完善脱贫激励机制，引导群众摒弃“等靠要”思想，激发贫困群众脱贫致富的信心、决心和热情，充分发挥贫困群众自主脱贫的主体作用。鼓励社会力量参与扶贫，广泛凝聚脱贫攻坚合力。

（八）持续保障改善民生，着力共创更好生活。始终把人民利益摆在至高无上的地位，不断满足人民日益增长的美好生活需要。促进社会就业和居民增收。落实就业优先战略和积极就业政策，注重解决结构性就业矛盾，解决好性别歧视、身份歧视问题。加强职业技能培训，鼓励创业带动就业。提升公共就业服务水平，促进高校毕业生等青年群体、农民工多渠道就业创业，加强零就业家庭、残疾人、低保对象等困难群体的就业援助，带动城乡居民收入较快增长。建立健全解决拖欠农民工工资问题的长效机制，严厉打击恶意欠薪行为。不断提高社会保障水平。完善城镇职工基本养老保险和城乡居民基本养老保险制度。做好城乡居民医疗保险整合及补充工伤保险试点工作，扩大医保异地结算范围。统筹城乡社会救助体系，完善最低生活保障制度。加强新形势下优抚安置工作，稳步推进“退役军人之家”建设，切实维护退役军人合法权益。扎实做好农村留守儿童、留守老人关爱保护工作。抓实农村敬老院提质改造三年行动计划。完成市救助站搬迁及残疾人康复中心项目建设。积极应对人口老龄化，发展老龄事业和养老产业，加快推进市康养中心建设。发展住房租赁市场特别是长期租赁，保护租赁利益相关方合法权益，支持专业化住房租赁企业和机构发展。加强公共租赁等保障性住房建设管理。全面推进社会事业发展。坚持教育优先发展，全力推进中心城区教育扩容、教育信息化建设、农村标准化学校建设、农村薄弱学校改造，促进县域内城乡义务教育一体化发展，着力解决中小学生课外负担重、“择校热”“大班额”等突出问题。科学规划和建设高中阶段学校，着力提升高中阶段教育普及率。紧密切合郴州产业发展需求，推进职业教育特色发展。加大公办幼儿园建设投入，办好学前教育，解决好婴幼儿照护和儿童早期教育服务问题。重视特殊教育、网络教育、继续教育，推进各类教育协调发展。鼓励社会力量兴办教育。持续推进校园周边环境综合整治。全面深化新时代教师队伍建设改革。加强青少年校外活动阵地建设。落实“健康郴州2030”规划。深化医药卫生体制改革，加强基层医疗卫生服务体系和贫困村卫生室标准化建设，完善基层医疗卫生机构补偿新机制，开展全科医生特设岗位工作，完善家庭医生签约服务，继续解决好“看病难、看病贵”问题。加强出生缺陷防治。深入开展爱国卫生运动，倡导健康文明生活方式。加强传染病防治，预防控制慢性非传染性疾病。积极支持中医药事业传承和发展。支持和规范社会资金进入养老、医疗等领域。深化计生服务管理改革，促进生育政策和经济社会政策配套衔接。抓好现代公共文化服务体系建设，扩大基层公共文化体育设施覆盖面，推进文化强市建设。继续抓好创建“体育之城”工作。加快推进小埠国家级运动休闲特色小镇建设。加强优秀文化传承和文化遗产保护。办好湘南起义90周年纪念活动。完成“村村响”市级播控平台建设，实现全市“村村响”“户户通”全覆盖和有线电视高清化。广泛开展全民阅读，抢占和拓展互联网思想文化舆论阵地。支持和发展志愿服务组织。夯实统计基层基础，提高统计工作水平。做好第四次全国经济普查工作。认真做好广播影视、新闻出版、民族宗教、外事侨务、贸促、海关、检验检疫、文物、档案、气象、地震、水文、科普、地方志、慈善和妇女儿童、老年人、残疾人等工作。加强和创新社会治理。完善社会治理体制机制和社会治安立体防控体系，巩固“平安郴州”创建成果。加强防范和处理邪教工作。着力解决网上虚假信息诈骗、倒卖个人信息等突出问题。继续抓好城乡社区综合服务平台建设。开展无上访乡镇（街道）、村（社区）创建活动，强化矛盾纠纷源头治理，推动依法及时就地解决信访问题。深入开展“五到五严五真五个一批”安全专项行动，持续深化安全生产大检查大整治，全面落实重大事故隐患治理“一单四制”，确保安全生产形势持续稳定向好。加强运输市场综合管理。确保车辆超限超载率稳控在0.1%以内。加强食品药品监管，严厉打击食品药品违法行为，打造“从田间到餐桌”“环保米粉”等放心食品工程。加强防灾减灾救灾和应急救援能力建设。落实消防工作责任，加强消防工作基层基础建设，攻坚克难全力整治市城区市场消防安全隐患。加强国家安全、国防动员、国防教育、人民防空、双拥共建工作，支持军队改革和驻郴部队建设，推进军民融合发展，争创全国双拥模范城。扎实抓好“十大惠民工程”。坚持积极有为、量力而行，继续办好一批民生实事，加快补齐影响群众生活品质的短板。①扶贫惠民工程：实现12.5万人脱贫；建档立卡贫困人口住院就医费用医保报销比例提高10%、大病保险起付线降低50%。②教育惠民工程：建设义务教育标准化教学点71所、农村公办幼儿园12所；新增城区学校学位1万个；补充乡村教师1500名。③健康惠民工程：农村适龄妇女“两癌”免费检查6.95万人、孕产妇产前筛查项目3.56万人；14万人参加职工医疗互助活动。④就业惠民工程：新增城镇就业6万人。⑤安居惠民工程：实施农村危房改造1.05万户；新增社会治安视频监控500个。⑥环境惠民工程：新建或改扩建公厕500座以上、改建农村户厕3万户以上；建设县城污水管网49公里，城镇污水处理达标率95%以上；继续抓好城乡垃圾转运体系和公（游）园管护；建设美丽乡村创建村11个、洁净乡村示范村50个、规范建房示范点11个；整村推进17个水库移民美丽家园建设。⑦供水惠民工程：解决农村14.46万人的安全饮水问题；建设（改造）或疏浚县城供水管网52公里。⑧交通惠民工程：提质改造农村公路700公里、建设安保设施750公里；自然村通水泥（沥青）路1031公里；危桥改造30座。⑨能源惠民工程：改造升级农村电网40个村；新增管道燃气用户2.5万户。⑩服务惠民工程：开展集贸市场和乡镇社区医疗机构计量器具免费检定；建设蜂窝物联网300个；改造农村宽带网络33个村。

（九）强化忠诚干净担当，着力加强自身建设。坚持党对一切工作的领导，加强“六抓”工作，努力建设忠诚、法治、办事、高效、廉洁的人民满意政府。坚定正确政治方向。深入学习贯彻党的十九大精神，以习近平新时代中国特色社会主义思想指引政府一切工作。牢固树立“四个意识”，严守政治纪律和政治规矩，坚决同以习近平同志为核心的党中央保持高度一致，坚决维护党中央权威和集中统一领导。扎实开展“不忘初心、牢记使命”主题教育。充分发挥全市行政学院（校）培训轮训公务员主渠道作用，提高政府公职人员“八项本领”。坚持严格依法行政。按照职能科学、权责法定、执法严明、公开公正、廉洁高效、守法诚信要求，全面推进依法履职、依法用权。做好政府立法工作，切实优化制度供给。落实行政决策程序规定和政府法律顾问制度，进一步提升政府依法科学民主决策水平。落实行政首长出庭应诉制度，做好行政应诉工作。自觉接受党内监督、人大监督、政协监督、国家监察机构监督、司法监督、舆论监督和社会监督。重大事项事先依法向人大报告，接受人大预算联网监督，认真研究处理市人大及其常委会交办的有关审议意见。进一步提高办理人大代表建议、政协提案质量。加强与各民主党派、工商联和各人民团体、社会各界人士的沟通联系。严守廉洁从政规定。坚持把纪律和规矩挺在前面，持之以恒加强作风建设和廉政建设。发扬钉钉子精神，从领导机关、领导班子和领导干部做起，以身作则、以上率下，强化监督检查，严肃执纪问责，坚决铲除腐败和不良作风滋生的土壤。以永远在路上的恒心和韧劲，一如既往地贯彻落实好中央八项规定精神，全力推动形成干部更加清正、政府更加清廉、政治更加清明的良好政治生态。持续推进作风建设。扎实开展“工作落实年”活动，弛而不息纠“四风”、转作风，坚决落实党中央、国务院和省委、省政府、市委的决策部署，以实际行动确保党的政令畅通、决策落地生根。深化政务公开，全面推行政务服务“一次办结”。开展减证便民活动，全面清理各种证明和手续，切实方便群众办事。对事关群众切身利益的重点领域的突出问题，采取过硬措施，扎扎实实抓好整改。

各位代表！新时代赋予新使命，新征程孕育新希望。让我们紧密地团结在以习近平同志为核心的党中央周围，在省委、省政府和市委的坚强领导下，在市人大、市政协的监督支持下，不忘初心、牢记使命，锐意进取、扎实苦干，为加快建设“五个郴州”、打造湖南“新增长极”、率先全面建成小康社会而努力奋斗！
